# Xylem versus phloem in secondary growth: a balancing act mediated by gibberellins

**DOI:** 10.1093/jxb/erab148

**Published:** 2021-05-04

**Authors:** Annelie Carlsbecker, Frauke Augstein

**Affiliations:** Department of Organismal Biology, Physiological Botany, Linnean Centre for Plant Biology, Uppsala University, Ullsv. 24E, SE-756 51, Uppsala, Sweden

**Keywords:** Arabidopsis, auxin, cambium, DELLA, hypocotyl, gibberellic acid, xylem expansion phase

## Abstract

This article comments on:

**Ben-Targem M, Ripper D, Bayer M, Ragni L. 2021**. Auxin and gibberellin signaling cross-talk promotes hypocotyl xylem expansion and cambium homeostasis. Journal of Experimental Botany **72**, 3647–3660.

**Secondary growth generates wood, which constitutes most of the plant biomass. Despite considerable efforts over the last decade to uncover the genetic and molecular regulation of the vascular cambium, there is still much to learn about how it produces wood (xylem) inward and bast (phloem) outward. Ben-Targem *et al.* (2021) now provide novel insight into how the hormones auxin and gibberellic acid (GA) govern the activity of the cambium, promoting a transition from formation of equal amounts of xylem and phloem to a stage where xylem formation dominates phloem in Arabidopsis hypocotyls, resembling wood formation in trees.**

Secondary growth is initiated when procambial cells between primary xylem and phloem begin to divide to form cambium that gives rise to more xylem and phloem, which increases organ girth. The secondary xylem supports the plant and provide means for water transport, whereas the phloem allows for sugar and signaling molecule translocation. In eudicots and gymnosperms, secondary growth is initiated in stems, roots, as well as in the hypocotyl, the embryonic stem joining the root with the stem (reviewed by Ragni and Greb, 2017). While the root and stem grow continuously, forming a developmental series from apical meristem towards the hypocotyl, the hypocotyl instead only grows radially with time, integrating signals from shoots and roots. In the Arabidopsis hypocotyl and upper root, secondary growth can be divided into two phases. The first phase is characterized by an equal production of secondary phloem and xylem, and the xylem contains vessels and parenchymatous cells. The second so-called xylem expansion phase follows the floral transition, and entails a shift towards production of relatively more xylem where fibers form in place of the parenchyma cells, similar to angiosperm wood. Key questions in research on secondary development focus on the mechanisms that underlie the initiation and maintenance of the vascular cambium and how shifts in the activity rate of the cambium are controlled to determine the relative amounts of xylem versus phloem produced. This is relevant for the hypocotyl, but also for seasonal growth dynamics or growth changes upon stress in trees. The study by Ben-Targem *et al.* (2021) now shows that increased GA levels upon flower induction trigger the activation of AUXIN RESPONSE FACTOR6 (ARF6) and ARF8 to promote xylem expansion. Ben-Targem *et al.* (2021) also show that ARF6 and ARF8 are critical for cambium initiation and formation of early secondary xylem. Hence, these findings contribute to our knowledge on fundamental questions in secondary growth research, but also open up interesting new questions worthy of further examination.

## Initiation and modulation of cambium activity

Recent research has drastically enhanced our understanding of how cambium is initiated and maintained in Arabidopsis (Box 1), recently reviewed by Fischer *et al.* (2019). Although quite a large number of signals and transcriptional regulators have been identified to act in the cambium, only a handful display phenotypic effects when mutated, indicating that the cambium is strongly buffered by redundantly acting players (Zhang *et al.*, 2019). Loss of cambium activity essentially only happens when both the *WOX4* and the *KNAT1/BREVIPEDICELLUS* (*BP*) genes are mutated. Hence, the new finding by Ben-Targem *et al.* (2021) that mutations in *ARF6* and *ARF8* in combination with *KNAT1/BP* also result in close to complete loss of cambium formation contributes potentially new and important players for the initiation and maintenance of the cambium.

In the transition to the xylem expansion phase, the hormone GA plays the lead role (Box 1). In rosette plants, such as Arabidopsis, initiation of the xylem expansion phase is preceded by bolting, the transition to flowering (Sibout *et al.*, 2008). This generates a graft transmissible signal, and Ragni *et al.* (2011) showed that this signal is GA. When GA is sensed by its receptor, DELLA proteins become targeted for degradation, releasing any protein bound by the DELLAs (Davière and Achard, 2016). In the context of the xylem expansion phase, DELLAs release their suppression of KNAT1/BP on the xylem side of the cambium. KNAT1/BP then are free to promote fiber formation (Felipo-Benavent *et al.*, 2018).

Because DELLAs bind a large set of different proteins, they also constitute a means to integrate GA signaling with signaling by other hormones (Davière and Achard, 2016). In the context of hypocotyl elongation, DELLAs regulate ARF6 and ARF8 (Oh *et al.*, 2014). The notion that DELLAs can bind ARF6 and ARF8 prompted Ben-Targem *et al.* to address the relevance of these factors for xylem expansion phase regulation.

## Xylem expansion phase is triggered when ARF6 and ARF8 are released from DELLAs

DELLA proteins are important negative regulators of the xylem expansion phase, as shown by their regulation of KNAT1/BP, which is critical for fiber differentiation (Felipo-Benavent *et al.*, 2018). Ben-Targem *et al.* (2021) identified the DELLA proteins GAI and RGA, primarily active in the phloem and cambium, as the main regulators of the xylem expansion phase. While the activity in the cambium probably affects KNAT1/BP, their activity in the phloem is paralleled by the expression of *ARF6* and *ARF8*, suggesting that they are important for the development of this tissue. Indeed, loss of function of these ARFs resulted in increased phloem divisions and formation of phloem fibers, but also in reduction of the xylem area. These phenotypes are similar to effects of expressing dominant DELLA mutants insensitive to GA degradation. Further supporting that the DELLAs act via ARF6 and ARF8, mutations in these genes prevented the enhanced xylem area that normally results from exogenous application of GA (Ben-Targem *et al.*, 2021). Hence, this suggests that upon elevated GA levels, ARF6 and ARF8 along with KNAT1/BP are released from inhibition by GAI and RGA. While KNAT/BP promotes fibers, ARF6 and ARF8 suppress phloem proliferation and differentiation, thus shifting the balance towards xylem vessel and fiber production. Together, these data reveal novel functions for ARF6 and ARF8 in secondary development, and show how auxin and GAs can crosstalk to regulate the xylem expansion phase. How ARF6 and ARF8 accomplish these feats is still unclear, but it is conceivable that they do so either as an indirect consequence of their effect on the phloem, or via non-cell-autonomous radial signaling.

Box 1. **Genetic and molecular regulation of the two phases of secondary growth**

(1) Secondary growth is initiated as auxin defines immature xylem cells as cambial organizers. These organizers induce stem cell activity in neighboring cells (Smetana *et al.*, 2019). The stem cells replenish the organizers as these differentiate into xylem on the inner side, and provide cells differentiating to phloem on the other side. Auxin acts via ARF5/MONOPTEROS (ARF5/MP) to active ATHB8, a HD-ZIP III transcription factor, which suppresses cell division and promotes xylem development. Auxin also induces expression of the transcription factor WOX4, which promotes cell division in the stem cells, as well as the receptor kinase PHLOEM INTERCALATED BY XYLEM (PXY). CLE peptides emanate from the phloem and are sensed by the PXY receptor to assert proper patterning over the cambium, and further promote WOX4 expression (Etchells *et al.*, 2016; Smit *et al.*, 2020). WOX4 together with KNAT1/BP are key cambial regulators, suggesting that the PXY–WOX4 and KNAT1/BP pathways operate synergistically and are fundamental for cambium activity (Zhang *et al.*, 2019). Ben-Targem *et al.* (2021) now also identify ARF6 and ARF8 as critical cambial activators together with KNAT1/BP. (2) The xylem expansion phase follows upon bolting when GA levels rise, resulting in the degradation of DELLA proteins releasing KNAT1/BP to promote fiber development and the xylem expansion phase (Felipo-Benavent *et al.*, 2018) and ARF6 and ARF8 to suppress phloem proliferation and differentiation, thereby enhancing xylem formation (Ben-Targem *et al.*, 2021).

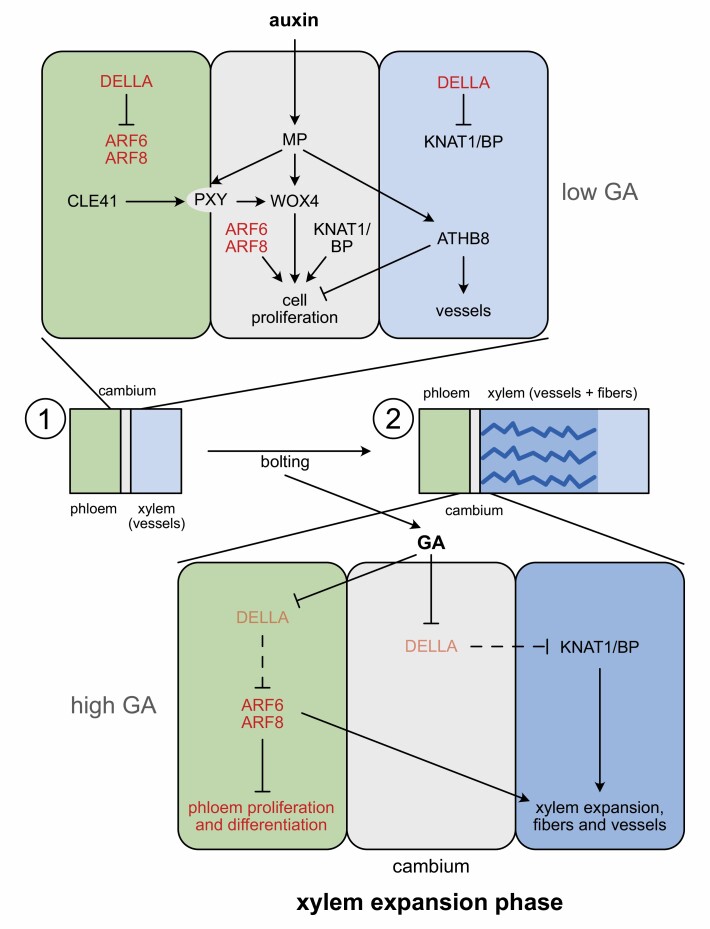

## On trees and weeds

Climate change has generated a strong need for efficient sequestration of carbon dioxide, and the woody biomass of trees is outstanding in its ability to store carbon (Pan *et al.*, 2011). Hence, how wood is formed and also how external conditions, such as drought and higher temperatures or mechanical stress, may affect tree growth are essential research areas. The study by Ben-Targem *et al.* (2021) emphasizes GA and its crosstalk with KNAT1/BP and ARF6 and ARF8 as critical input for the Arabidopsis hypocotyl xylem expansion phase. Could this finding be of general significance for non-rosette plants and even for trees? Indeed, GAs are also important in trees for promotion of xylem development and fiber formation (Eriksson *et al.*, 2000; Mauriat and Moritz, 2009; Gou *et al.*, 2011) and are implicated in tension wood formation upon mechanical stress (Gerttula *et al.*, 2015). Furthermore, a considerable crosstalk between GA and auxin signaling has been documented in *Populus* wood development (Björklund *et al.*, 2007; Johnsson *et al.*, 2019).

GA signaling and, in particular, DELLA function have been implicated in various abiotic stresses. Cold, salt, and drought stress lead to lowered levels of active GA and consequently enhanced DELLA activity (reviewed by Colebrook *et al.*, 2014). In seeds, signaling via the stress hormone abscisic acid (ABA) also tightly integrates with GAs and DELLAs (Lim *et al.*, 2013). As ABA affects xylem development during both primary and secondary growth (Campbell *et al.*, 2018; Ramachandran *et al.*, 2018), it is possible that GA and ABA signaling also integrate here to balance growth and stress responses. Future research will reveal how regulation of secondary growth and xylem expansion is wired to sustain growth under optimal and adverse conditions, but it is clear that the new pieces to the puzzle of secondary growth regulation introduced by Ben-Targem *et al.* (2021) open up the way for additional pieces to be put into place.
